# The effectiveness of vaccination, testing, and lockdown strategies against COVID-19

**DOI:** 10.1007/s10754-023-09352-1

**Published:** 2023-04-27

**Authors:** Marlon Fritz, Thomas Gries, Margarete Redlin

**Affiliations:** Department of Economics, Paderborn University, Warburger Str. 100, 33098 Paderborn, Germany

**Keywords:** Pandemics, COVID-19, Economics, Vaccination, Testing, Non-pharmaceutical interventions, Effectiveness, I18, C23

## Abstract

The ability of various policy activities to reduce the reproduction rate of the COVID-19 disease is widely discussed. Using a stringency index that comprises a variety of lockdown levels, such as school and workplace closures, we analyze the effectiveness of government restrictions. At the same time, we investigate the capacity of a range of lockdown measures to lower the reproduction rate by considering vaccination rates and testing strategies. By including all three components in an SIR (Susceptible, Infected, Recovery) model, we show that a general and comprehensive test strategy is instrumental in reducing the spread of COVID-19. The empirical study demonstrates that testing and isolation represent a highly effective and preferable approach towards overcoming the pandemic, in particular until vaccination rates have risen to the point of herd immunity.

## Introduction

The global COVID-19 outbreak has created a wide range of responses from governments. The introduction and subsequent effect of government policies are subject to much debate even in recent times where most governments started to re-open and relax most of the restrictions. These policies vary between countries independently of their level of economic and social development. While the ability of lockdowns and vaccination schemes in order to curb the spread of COVID-19 is undisputed, little is known about the detailed effect of different lockdown strategies on the spread of COVID-19 and its interaction with widespread testing. Following (Kerr et al., 2020) testing and quarantine strategies are as important as vaccinations, especially in the short run, and so the importance of a broad testing strategy is very much a subject of public debate. Furthermore, the recent development of new variants of COVID-19 and a stagnating vaccination rate also demonstrate the importance of testing and lockdown strategies in the long run, which are further discussed in the literature section. Additionally, it must be noted that population-scale testing strategies are also important because they provide important insights into the development of the pandemic (Mercer & Salit, 2021). They provide critical viral prevalence data to steer our response to the pandemic, allow measurement of the rate of virus spread in a population and helps identify regional hotspots and high-risk subpopulations.

In this paper we analyze the effects of government decisions in 37 OECD countries, distinguishing between different degrees of lockdown, e.g., the shutdown of schools, restaurants, sport clubs, and other social activities. We also look in detail at the impact of vaccinations on the change in the reproduction rate. Finally, we analyze the impact of testing on the decline in the reproduction rate. Although (Ge et al., 2021) disentangle the interplay between policies and vaccination, the effect of test strategies on the spread of COVID-19 remains an open topic. To shed more light on the effectiveness of testing, government restrictions, and vaccination, we simultaneously analyze the influence of all three measures on the spread of COVID-19, specifically on the reproduction rate. Thus, we extend the analysis of Ge et al. (2021) who analyze the effects of non-pharmaceutical interventions (NPI) and vaccinations by including a detailed test strategy in their investigation. Therefore, we extend the standard SIR model as described in Cherif and Hasanov (2020) or (Avery et al., 2020) by allowing for the influence of testing, lockdowns, and vaccination. We propose that a test strategy in combination with vaccination is both an effective and economically useful approach to reduce the spread of COVID-19 in the long run.

The results of our baseline regression indicate that testing, vaccinations, and stricter policy measures, as expected, are associated with a significant reduction in the reproduction rate. A comprehensive test strategy is particularly effective in reducing the reproduction rate, since a one-unit increase in daily testing (e.g., from the mean value 3.22–4.22 tests per one thousand people) has a similar effect as increasing the vaccination rate by 3%. Our findings help to explain cross-country differences in COVID-19 spreading. Combined social distancing and testing strategies in the early phases of the epidemic are demonstrated to be more efficient at reducing the disease burden, and they can delay the peak of the disease.

Whereas recent papers consider non-pharmaceutical interventions in isolation (Ge et al., 2021; Kerr et al., 2020), largely disregarding other instruments, our analysis looks at stringency measures in context with other policy measures and hence provides a more comprehensive picture. The detailed analysis of various components of the lockdown stringency index shows that school and workplace closures have the strongest (significant) effects. Nevertheless, they come with major social and economic costs; what is more, especially the long-run consequences of school closures and other social distancing measures are unpredictable. Therefore, for as long as vaccine doses are not available in sufficient quantity or vaccinations are not embraced by the population, a “test, trace, and isolate” strategy can help to keep the pandemic under control without having it continuing to influence our lives in unknown ways.

The remainder of this paper is organized as follows. Sect. “Literature“ contains an overview of recent studies with a focus on empirical findings. Sect. “Theoretical model“ introduces the standard SIR model, which comprises policies, vaccinations, and testing strategies. Sect. “Estimation strategy“ presents the transition from theory to the empirical model. Sect. “Empirical evidence“ shows empirical evidence, and Sect. “Concluding remarks“ concludes.

## Literature

There is a large and growing body of literature on the effects of COVID-19 ranging from individual health analyses to aggregate economic studies. Nevertheless, some effects remain uninvestigated. (Hartl et al., 2020) analyze the effect of lockdowns in Germany using data from Johns Hopkins University. The data are advantageous in that they match various statistics, i.e., from Germany’s Robert Koch Institute and the WHO. The authors seek to estimate the effect of different policies by identifying a trend break in the cumulated number of confirmed cases of COVID-19 after March 20, 2020. Specifically, they identify a drop in the growth rate of reported cases from 26.7 to 13.8% after March 20, 2020. The German government introduced new policies on March 13, 2020, so there is an expected 7-day lag which can be split into 5 days for the incubation period (Lauer et al., 2020; Linton et al., 2020), plus up to 2 days until testing, and an additional 1 or 2 days until the case is reported (Hartl et al., 2020).

Alfano and Ercolano (2020) also argue for the effectiveness of lockdowns in reducing the reproduction rate. In contrast to Hartl et al. (2020) the authors use a panel data set with a lockdown measure for 100 countries. Their results show that lockdowns are able to reduce the number of COVID-19 cases. They distinguish between two different policy strands: (i) health policies and (ii) policies aimed at reducing the spread of COVID-19. Usually, the latter are characterized by high economic costs. Consequently, the economic debate focuses on the “trade-off between safeguarding citizens’ health and avoiding damage to the economy” [(Alfano & Ercolano, 2020), p. 510; see also Goolsbee and Syverson 2021]. However, this debate is restricted to single countries, a restriction that is relaxed within our analysis. We want to disentangle the effect of different lockdown policies to determine the optimal trade-off between safeguarding individuals and reducing the cost to the economy.

Ashraf (2020) finds a similar negative effect of government-imposed social distancing on the number of confirmed cases. However, he focuses on the impact on stock markets and shows positive effects of testing and quarantining on market returns. Jarvis et al., (2020) show that lockdown policies decrease the reproduction rate from 2.6 to 0.62.

Askitas et al., (2020) analyze the effects of a range of policy activities in more detail by proposing that the spread of COVID-19 is influenced by environmental, behavioral, and social factors. They investigate the effect of type and intensity of policies on daily incidence rates. Their results show that cancelling public events and imposing restrictions on private gatherings are the most efficient strategies. School closures, too, have a strong influence on the daily incidence of COVID-19. Surprisingly, workplace closures and stay-at-home requirements are not significant.

Cherif and Hasanov (2020) propose that besides lockdowns and isolation, testing is another economically preferable strategy for curbing the spread of COVID-19. In their theoretical study, strategic group testing and periodic testing are shown to be effective in overcoming the pandemic. Also (Baldwin, 2020) strongly recommends a massive increase in testing capacities. He points out that besides vaccinations, testing is a way “to isolate the sick from the healthy” [(Baldwin, 2020), p.1]. (Taipale et al., 2020) show that testing is effective at any stage of the pandemic and can be used in addition to other curbing strategies such as partial lockdowns. In other words, the number of individuals who are identified and isolated matters to the development of the pandemic [(Taipale et al., 2020), p. 2]. The authors propose that ideally, almost everyone should undergo appropriate PCR testing in order to reduce the reproduction rate to below 1. Moreover, (Taipale et al., 2020) argue that a testing strategy has an advantage over a general lockdown since the population and the economy can accept many false-positive tests. The number of false-positive tests is in any case lower than the false-positive rate during a general lockdown. Although some evidence has been provided in simulation studies, empirical studies on the influence of testing on the reproduction rate are missing so far. Kerr et al., (2020) also propose to increase testing levels by conducting a simulation exercise for the Seattle metropolitan area. They find that effective isolation and routine testing are capable of reducing the reproduction rate. Nevertheless, (Kerr et al., 2020) warn that a testing strategy is only effective in curbing the spread of COVID-19 when case numbers are relatively low.

The final and possibly most important driver of the reproduction rate in the long run is vaccination. Although other variants of COVID-19, as for example the Omicron variant may have an influence, they are not explicitly modelled since vaccination also weaken their effect. Although the ability of vaccinations to curb the spread of COVID-19 is undisputed, estimates concerning the threshold in order to achieve herd immunity are rather inconclusive. Ke et al., (2021) estimate the threshold for herd immunity to be between 71 and 84%. They recommend encouraging the population to get vaccinated and advocate for more public education so herd immunity can be achieved. Moreover, the study demonstrates that strong control measures are a driver when it comes to curbing the spread of COVID-19. Further (Fontanet & Cauchemez, 2020) use a more optimistic threshold by proposing herd immunity if 50% of a population are immune. Nevertheless, looking at current numbers out of the UK it is clear that this threshold is too low. Ge et al., (2021) analyze the interaction between testing and vaccination in 133 countries across different waves. Although vaccination has a growing effect in reducing the spread of COVID-19, NPIs are the most effective measures so far. Furthermore, new variants and their possible resistance to vaccinations reinforce the debate on NPIs. For example, Germany has a vaccination rate around 75% (March 2022), which makes an argument for the importance of other effective policies. Since new variants and their influence cannot be forecasted, recent literature proposes results “that policymakers and individuals should consider maintaining non-pharmaceutical interventions and transmission-reducing behaviours throughout the entire vaccination period” [(Rella et al., 2021), p.1]. Moreover, frequent large-scale testing should remain part of strategies to contain COVID-19 since it can substitute for many non-pharmaceutical interventions that come at a much larger cost to individuals, society, and the economy (Gabler et al., 2021).

One constraint of existing studies is country differences, since population density, demographic factors, and weather play important roles. (Ge et al., 2021) conclude that vaccination is the most promising way out of this pandemic when accessible in all countries equally. Their study demonstrates that the cumulative effectiveness of vaccination increases. In other words, it is important to understand that individuals acquire the desired level of immunity 12 days after their first dose, and that the effect of vaccination grows exponentially as herd immunity is approached.

In this paper we extend the analysis of Ge et al. (2021) by including testing strategies in our investigation of NPIs and vaccination. Therefore, we extend the standard SIR model as described in Cherif and Hasanov (2020) or (Avery et al., 2020) to allow for the impact of testing, lockdowns, and vaccination. The empirical analysis rounds off our theoretical considerations.

## Theoretical model

To empirically examine the effectiveness of policies that can and indeed are used to curb the spread of the disease, we first need to identify them in a theoretical model. We depart from a standard epidemiological model such as the SIR (Susceptible, Infected, Recovery) model as discussed by, e.g., (Cherif & Hasanov, 2020) or (Avery et al., 2020). In this model, individuals are in one of these three states at any given time. Given a total population of N, the number of susceptible S(t) individuals, the number of infected I(t) individuals and the number of recovered R(t) individuals adds up to S(t) + I(t) + R(t) = N. With an infection rate β and a recovery rate γ and assuming that N = 1, we can rewrite this standard SIR model of epidemic dynamics as

$$\dot{S}\left( t \right) = - \beta S\left( t \right)\frac{{I\left( t \right)}}{N}$$

$$\dot{I}\left( t \right) = \beta S\left( t \right)\frac{I\left( t \right)}{N} - \gamma I\left( t \right)$$

$$\dot{R}\left( t \right) = \gamma I\left( t \right).$$

The SIR model is a basic model. There are a number of variations and extension of the SIR model. E.g., after recovery from an infection individuals may have only temporary immune protection. In such a case the model would turn into another class of models, the SIRS model (Susceptible, Infected, Recovered, Susceptible). However, for the virus variation which we consider in this paper this scenario is seemingly not the case.

### Modelling policy effects on the effective reproduction rate

An additional important characteristic of any epidemic is the basic reproduction rate RR(0), which indicates how many other people an infected individual infects under natural starting conditions. Thus, the basic reproduction rate is akin to a descriptor of the natural dynamics of a disease. In the SIR model, the basic reproduction rate is the “natural ratio” of newly infected people to one infected person per unit of time $\beta$ divided by the simultaneous recovery rate $\gamma$ per unit of time

$${\text{RR}}\left( 0 \right) = \frac{\beta }{\gamma }$$

Without policy interventions, a disease will naturally spread if the ratio of newly infected to recovering individuals is larger than one ($RR(0)>1$). If $RR(0)<1$, the disease will disappear because every day more individuals recover than contribute to a further spread. As we are interested in policies that curb the spread of a disease from the start, we need to develop a view of this model that allows for a discussion of effective policy measures. As the reproduction rate indicates if a disease spreads (RR > 1), stagnates (RR = 1) or declines (RR < 1), the reproduction rate is a major indicator of the epidemic dynamic. Thus, we use the reproduction rate $RR$ to discuss the infection process and potential policy interventions in detail. To do this, we depart from the basic reproduction rate and use the effective reproduction rate. The effective reproduction rate defines a time dependent reproduction rate when certain policies are introduced and a fraction of the host population that is susceptible is included.^1^ For a stylized discussion of measures to control the spread of the disease we look at the *effective reproduction rate* as

$${\text{RR}}\left( t \right) = \frac{{p_{\kappa } *\kappa }}{\gamma }p_{S} \left( t \right)$$

In this equation the rate of newly infected individuals $(\beta ={p}_{\kappa }*\kappa )$ is explained by $\kappa ,$ the average number of potentially infectious contacts of an infected individual, the probability of transmission with each contact ${p}_{\kappa }$. With ${p}_{S}=\frac{S\left(t\right)}{N}$ we describe probability that a contact is a susceptible individual. Thus, we can rewrite

$${\text{RR}}\left( t \right) = \frac{{p_{\kappa } \left( t \right)*\kappa \left( t \right)}}{\gamma }\frac{S\left( t \right)}{N}$$

With the help of this equation we can examine the effects of policy measures. In the empirical section we examine three of the following four policies: (1) distancing rules, (2) degrees of lockdowns, (3) testing and quarantine strategies, and (4) vaccination strategies.

1. To motivate the first two (and also most common) measures, we can look at the parameters ${p}_{\kappa }$ and $\kappa$ in more detail. Standard measures to reduce the probability of transmission are social distancing and protective measures such as masks. Therefore, the original probability of transmission ${p}_{\kappa 0}$ is reduced by the degree of the distance rule $d(t)$ which holds at time t, such that we obtain
  $$p_{\kappa } \left( t \right) = p_{\kappa 0} \left( {1 - d\left( t \right)} \right)$$

Note that d(t) simple depicts the effect of distance measures on the probability of transmission. Different measure may have even different and even non-linear effects on this probability. E.g., wearing a simple textile mask may already have an effect on the transmission of the virus. However, with a medical mask this transmission probability may further decline and with an FFP2 mask it may even improve exponentially. However, the result of these various is a reduction in transmission probability which we describe with parameter d(t).

(2) Another common policy measure is to reduce the number of contacts an individual can have. Without any policy, the average infected individual is characterized by ${\kappa }_{0}$ contacts. If the number of contacts is reduced, infected but as yet undetected individuals will have fewer contacts. Therefore, a standard policy instrument is to reduce the number of contacts via varying degrees of lockdown. Depending on the stringency of government actions at time t, the original number of contacts ${\kappa }_{0}$ can be reduced further by a certain percentage rate of contact reduction $c(t)$, such that
  $$\kappa \left( t \right) = \kappa_{0} \left( {1 - c\left( t \right)} \right).$$

As we will discuss in the empirical section, the reduction of contacts will be indicated by a stringency index.

(3) The next measure to reduce the number of contacts that spread the disease $\kappa$ is quarantining. If all individuals who are infected are immediately detected and quarantined, the number of infectious contacts reduces to zero and the disease cannot spread. However, this is not realistic, especially not in the case of COVID-19 given that some infected individuals do not show any symptoms. While symptomatic individuals can be identified and quarantined, asymptomatic individuals may not and hence continue to spread the virus. This risk can be reduced by implementing systematic testing and isolation. If an infected asymptomatic individual is tested positive and quarantined, they cannot spread the virus and hence contribute to the infection process. Therefore, the number really infected individuals that spread the disease can be reduced through this detection. How can these asymptomatic individuals who spread the virus invisibly be found? If the asymptomatic infected are distributed randomly in the total population, and $\tau (t)$ is the share of the total population per period that is tested, $\tau (t)$ is also the share of the asymptomatic infected individuals invisibly spreading the virus. These infected individuals can now be quarantined so they no longer contribute to the epidemic. So, the test activity reduces the total number of infectious contacts by rate $\tau (t)$—some infected individuals are detected and completely neutralized trough quarantine, and some are still fully contributing to the infectious dynamics. However, since the R value is about passing on the virus from an average infected, this effect of testing can be included in the R value as a reduction of the average number of contacts of an infected and still invisibly spreading individual. Thus, the remaining average number of contacts of an average infected and invisibly spreading individual is now
  $$\kappa \left( t \right) = \kappa_{0} - \tau \left( t \right)\kappa_{0} = \kappa_{0} \left( {1 - c\left( t \right)} \right)\left( {1 - \tau \left( t \right)} \right).$$

Thus, a general systematic testing per period would reduce the average number of contacts of an average infected individual.

(4) Vaccination is expected to not only protect the vaccinated individual, but also reduce or even completely eliminate the risk of further spreading. Vaccination affects the probability of an infected individual randomly meeting a susceptible individual, ${p}_{S}(t)=\frac{S(t)}{N}$. Thus, the number of susceptible individuals is reduced by the share $v(t)$ of vaccinated individuals at t. If $r\left(t\right)$ is the total share of recovered individuals and if we normalize the total number of individuals to one, we obtain
  $$p_{S} \left( t \right) = S\left( t \right)/N = \left( {1 - v\left( t \right)} \right)\left( {1 - r\left( t \right)} \right)$$

After this short and simplifying discussion of each type of policy, we now write down the entire mechanism that determines the effective reproduction rate:

$${\text{RR}}\left( t \right) = \frac{{p_{{\kappa 0}} \kappa _{0} }}{\gamma }\overbrace {{\left( {1 - d\left( t \right)} \right)}}^{{\begin{array}{*{20}c} {{\text{distance}}} \\ {{\text{policy}}} \\ \end{array} }}\overbrace {{\left( {1 - c\left( t \right)} \right)}}^{{\begin{array}{*{20}c} {{\text{lockdown}}} \\ {{\text{policy}}} \\ \end{array} }}\overbrace {{\left( {1 - \tau \left( t \right)} \right)}}^{{\begin{array}{*{20}c} {{\text{test}\,\&\,\text{quar}\,\text{policy}}} \\ \end{array} }}\overbrace {{\left( {{\text{1}} - {\text{v(t)}}} \right)}}^{{\begin{array}{*{20}c} {{\text{vaccination}}} \\ {{\text{policy}}} \\ \end{array} }}\left( {{\text{1}} - {\text{r(t)}}} \right)$$

If we assume that the rate of recovered individuals at the beginning of the process is close to zero, we see that the effective reproduction rate is the basic reproduction rate corrected by the policy instruments $RR(t)=RR(0)\left(\text{policy effect}\right)$. However, note that the four policies have different qualities. The only policy with a permanent effect after a once-and-for-all action is vaccination. The accumulated stock of vaccinations will permanently reduce the effective reproduction ratio; once herd immunity is reached, none of the other policies are needed. All other instruments are only effective for as long as they are applied. A lockdown only brings down the effective reproduction rate for as long as it remains in place. As soon as it is lifted, the infection dynamics start up again, as reflected in the basic reproduction rate. However, we also see that all policies are substitutes. A reduction in contacts per day through a lockdown is generally substitutable to systematic tests per day combined with a quarantine policy. The possibility to substitute one policy for another allows for an efficient policy choice. Thus, policy-makers can choose the cheapest policy instrument given the same reduction in infection dynamics.

## Estimation strategy

In the previous theoretical considerations we derived how policy measures potentially affect the effective reproduction rate. With the help of this modeling we can now motivate our estimation strategy and the particular estimation models. We derived four COVID-19 policy measures that potentially affect the reproduction rate. These are: (1) distance rules, (2) degrees of lockdown, (3) testing and quarantine strategies and (4) vaccination strategies. Since the exact policy measures and expected outcomes such as the number of contacts or the distance rule are difficult to measure directly, in the empirical model we proxy these measures by available variables. First, lockdown policy is represented by the stringency index which is a lockdown composite measure based on nine government response indicators and presents the non-pharmaceutical interventions. Unfortunately, to our best knowledge, there is no data available, which would allow to measure the distance rule for our country set and daily base. However, we believe that this is also indirectly determined by the non-pharmaceutical interventions included in the analysis. Second, test and quarantine policy is proxied by the number of COVID-19 tests carried out in the population. Finally, vaccination policy is represented by the vaccination rate in the population. Our starting point is a model where we estimate a linear combination of the policy measures

$$\Delta {\text{RR}}\left( t \right) = \alpha + \overbrace {{\beta _{1} ~{\text{stringenc}}~{\text{index}}_{{i,t - 1}} }}^{{\begin{array}{*{20}c} {{\text{lockdown}}~{\text{and}}~{\text{distance}}} \\ {{\text{policy}}} \\ \end{array} }}\overbrace {{ + \beta _{2} ~{\text{tests}}_{{i,~t - 1}} }}^{{\begin{array}{*{20}c} {{\text{test}\,\&\,\text{quar}\,\text{policy}}.} \\ {{\text{policy}}} \\ \end{array} }} + \overbrace {{\beta _{3} ~{\text{vaccinations}}_{{i,t - 7}} }}^{{\begin{array}{*{20}c} {{\text{vaccination}}} \\ {{\text{policy}}} \\ \end{array} }} + ~\mu _{i} + \varepsilon _{{i,t}}$$

where ${\Delta RR}_{i,t}$ is the change in the reproduction rate in country i at day t, ${tests}_{i, t-1}$ is the number of new COVID-19 tests per 1,000 people in country i at day t-1, ${vaccinations}_{i,t-7}$, is the number of people who received a vaccination per 100 people in country i at day t$-$7, ${stringenc index}_{i,t-1}$ is a composite government response indicator in country i at day t$-$1, and the disturbance term is composed of the individual effect ${\mu }_{i}$ and the stochastic disturbance ${\epsilon }_{i,t}$. We select the change in the reproduction rate as the dependent variable. This allows us to measure the effect of policy measures on reproduction rate dynamics while ignoring differences between countries. Furthermore, we avoid possible reverse causality between the policy measures and the reproduction rate.

In the further course we also estimate specifications including interactions of ${stringenc index}_{i,t-1}$ and ${tests}_{i, t-1}$ with the vaccination rate resulting in

$$\begin{gathered} \Delta {\text{RR}}_{i,t} = \alpha + \beta_{1} {\text{stringenc}}\, {\text{index}}_{i,t - 1} + \beta_{2}\, {\text{tests}}_{i, t - 1} + \beta_{3}\, {\text{vaccinations}}_{i,t - 7} \hfill \\ \quad \quad \quad \quad + \beta_{4}\, {\text{stringenc}} {\text{index}}_{i,t - 1} * {\text{vaccinations}}_{i,t - 7} + \mu_{i} + \varepsilon_{i,t} , \hfill \\ \end{gathered}$$

$$\begin{gathered} \Delta {\text{RR}}_{i,t} = \alpha + \beta_{1}\, {\text{stringenc}}\, {\text{index}}_{i,t - 1} + \beta_{2} {\text{ tests}}_{i, t - 1} + \beta_{3} \,{\text{vaccinations}}_{i,t - 7} \hfill \\ \quad \quad \quad \quad + \beta_{4}\, {\text{tests}}_{i, t - 1} * {\text{vaccinations}}_{i,t - 7} + \mu_{i} + \varepsilon_{i,t} . \hfill \\ \end{gathered}$$

These allow us to examine how the mix of COVID-19 measures behaves with an increase in the vaccination rate in the population.

## Empirical evidence

Our analysis is based on the COVID-19 dataset from the governmental organization Our World in Data which provides a daily updated collection of COVID-19 variables including confirmed cases, vaccinations, deaths, testing data and state stringency. The data of the stringency index provided by Our World in Data are based on the Oxford COVID-19 Government Response Tracker. We use a panel dataset of daily data covering 37 OECD countries from the period March 1, 2020 to May 20, 2021.^2^

### Dependent variable

Our dependent variable $RR$ represents the average number of new infections caused by a single infected individual. If the rate is greater than 1, the infection is able to spread in the population. If it is below 1, the number of cases occurring in the population will gradually decrease to zero. The data used is based on estimates of Arroyo-Marioli et al. (2021) and represents the real-time effective reproduction rate of COVID-19. The authors exploit the fact that in the benchmark SIR model RR is linearly related to the growth rate of the number of infected individuals. Using data on new cases to construct a time series of how many individuals are infected at a given point in time, they estimate the growth rate of this time series with the Kalman filter and leverage the theoretical relationship given by the SIR model to obtain R from the estimated growth rate. At this point it should be noted that the actual number of COVID-19 cases is not known. The number of confirmed cases is lower than the number of actual cases and depends on the testing strategy. However, (Arroyo-Marioli et al., 2021) show that their estimates are fairly accurate even when new cases are imperfectly measured.

### Explanatory variables

The independent variable $tests$ is defined as the daily number of tests for COVID-19 per 1000 people. Because the number of tests is often volatile from day to day, we use a seven-day rolling average. We should note here that the number of tests is based on country specific sources and does not refer to the same in each country. Although all country statistics are based on laboratory-conducted tests, the differences are that some countries report the number of people tested, while others report the number of tests performed which can be higher if the same person is tested more than once. Further, for most of the countries the test date is based on PCR tests only while in some countries also laboratory antigen tests are included.

We use two alternative variables for $vaccinations$. First, we use the total number of individuals who received at least one vaccine dose. The registration studies of vaccines show that the first vaccination is already effective after a few days. For this reason, the vaccination variable is included in the model with a lag of 7 days. Alternatively, we include the total number of individuals who received all doses prescribed by the vaccination protocol. Full protection can only be expected from about seven days after the second vaccination.

The $stringenc index$ is a composite measure based on nine government response indicators, namely school closures; workplace closures; cancellation of public events; restrictions on public gatherings; closures of public transport; stay-at-home requirements; public information campaigns; restrictions on internal movements; and international travel controls. The index on any given day is calculated as the mean score of the nine metrics, each taking a value between 0 and 100. A higher score indicates a stricter response (i.e., 100 = strictest response). In addition to the overall index, we also verify the influence of the individual index components. Table 1 presents the descriptive statistics of all variables. A detailed description of definitions and sources of all variables is available in Table 6 in the Appendix. During the observation period, the average reproduction rate was 1.09. The daily number of tests per 1000 individuals was 3.22, peaking at 63.46. It should be noted that for most of the countries only PCR tests performed in the laboratory are included in the statistics, disregarding rapid and self-tests. Since a PCR test is usually performed after a positive rapid or self-test and the positive rate of rapid tests is very low, the number of rapid and self-tests is many times greater than the official number of PCR tests. The proportion of vaccinated and fully vaccinated individuals was on average 12.67% and 6.37%, respectively. The differences between countries are relatively large. For example, while Israel and the UK had percentages of 62 and 54%, respectively, at the end of the observation period Australia and Japan were under 3%. The Stringency Index averaged 60.60, ranging from 0 (no restrictions) to 96.3 close to the maximum (highest restrictions).

**Table 1:** Descriptive statistics

Variable	Obs	Mean	Std. Dev	Min	Max
Reproduction rate	15,958	1.0904	0.3651	0.22	5.18
Reproduction rate (change)	15,921	− 0.0036	0.0371	− 0.74	0.67
Tests	15,197	3.2224	6.05992	0.001	63.46
Vaccinations	4,147	12.6880	13.6471	0	62.79
Vaccinations (fully)	3,703	6.3664	9.6143	0	58.95
Stringency index	16,338	60.5999	17.6081	0	96.3
School closures	16,344	1.9408	0.9247	0	3
Workplace closures	16,349	1.8593	0.8449	0	3
Cancelled public events	16,332	1.6410	0.6306	0	2
Restrictions on gatherings	16,339	3.1459	1.2641	0	4
Public transport closures	16,343	0.5139	0.5636	0	2
Stay-at-home requirements	16,343	1.1419	0.8546	0	3
Restrictions on internal movement	16,344	1.0010	0.8689	0	2
International travel controls	16,336	2.9183	0.8745	0	4
Public information campaigns	16,337	1.9652	0.2126	0	2

### Regression results

Table 2 presents the results of our baseline specification.^3^ The results indicate that testing, vaccinations, and stricter policy measures show, as expected, highly significant negative correlations with the dynamic of the reproduction rate. The simultaneous consideration of these instruments in our study is critical when it comes to understanding the effectiveness of these measures in the real world and under different conditions and strategies within countries.

**Table 2:** Fixed-effects results

	(1)	(2)	(3)	(4)	(5)	(6)
	ΔRR	ΔRR	ΔRR	ΔRR	ΔRR	ΔRR
Tests _t-1_	− 0.00041^***^ (− 6.180)	− 0.00124^***^ (− 12.629)	− 0.00037^***^ (− 5.419)	− 0.00110^***^ (− 10.742)	− 0.00040^***^ (− 5.182)	− 0.00041^***^ (− 6.086)
Vaccinations _t-7_	− 0.00016^***^ (− 5.435)		− 0.00029^***^ (− 4.811)		− 0.00016^***^ (− 4.380)	− 0.00015 (− 1.196)
Vaccinations (full) _t-7_		− 0.00034^***^ (− 6.696)		− 0.00068^***^ (− 7.510)		
(Vaccinations _t-7_)^2^			0.000003^**^ (2.436)			
(Vaccinations (full) _t-7_)^2^				0.000008^***^ (4.536)		
Stringency index _t-1_	− 0.00030^***^ (− 7.679)	− 0.00041^***^ (− 9.468)	− 0.00027^***^ (− 6.479)	− 0.00040^***^ (− 9.196)	− 0.00031^***^ (− 7.674)	− 0.00030^***^ (− 6.026)
Vaccinations _t-7_ * Tests _t-1_					− 0.00000 (− 0.225)	
Vaccinations _t-7_ * Stringency _t-1_						− 0.00000 (− 0.086)
*R*^*2*^* within*	0.028	0.066	0.030	0.072	0.028	0.028
*Countries*	37	36	37	36	37	37
*Obs*	3810	3366	3810	3366	3810	3810

*t* statistics in parentheses

*p < 0.10, **p < 0.05, ***p < 0.01

A permanent one-unit increase in daily testing (e.g., from the mean value 3.22–4.22 tests per 1000 individuals) has a similar effect as increasing the vaccination rate by 3%. This result is in line with previous studies which argue that testing, quarantine, and isolation strategies can be effective instruments for controlling the pandemic and that routine testing campaigns in combination with isolation as well as contact tracing and testing can decrease the infection rate (Kerr et al., 2020; Pavelka et al., 2020; Taipale et al., 2020). Our results go beyond general country studies and show in a broader data-based assessment that testing, even when other tools are taken into account, makes a significant contribution to the fight against the pandemic.

Increasing the stringency index by one unit (e.g., from recommended school closures to required school closures) has a similar effect as increasing the vaccination rate by 2%. When weighing up the costs of the individual measures, it should be borne in mind that while testing incurs permanent costs, vaccinations and the duration of immunity incur only one-off costs.

**Fig. 1:**
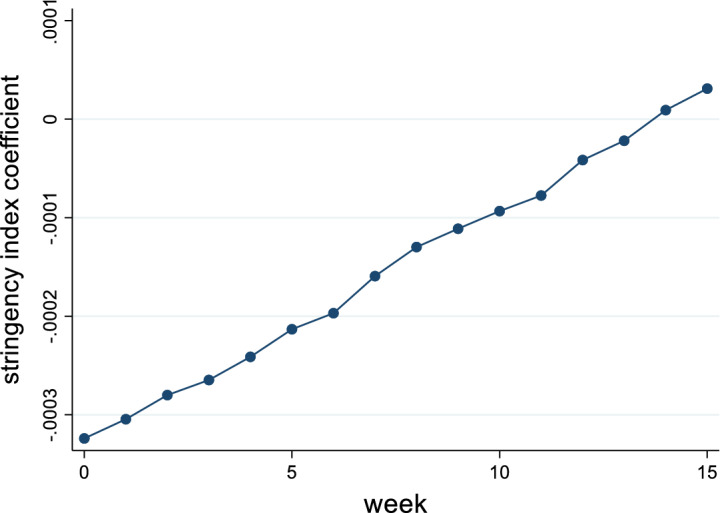
Effect of NPIs lagged by 1 to 15 weeks

To examine how the effect of social distancing changes over time, we estimate the coefficients for different lengths of time lag. Figure 1 shows how the effects behave for a lag of 1–15 weeks. In general, we see that the effect of an increase in the stringency index is strongest immediately, decreases with time and approaches zero over time. After 12 weeks the effect turns insignificant indicating that the adoption time of NPIs and the adjustment to current conditions is important. Responding early is essential to counteract the virus most effectively, since the effect is visible and strongest directly after introduction.

Next, we take a closer look at the vaccination effect. According to the vaccine manufacturers, maximum protection is only afforded after complete vaccination. Clinical person-level studies (Bernal et al., 2021; Thompson et al., 2021) estimate a mRNA vaccine effectiveness for prevention of infection of about 80% with the first dose and an increase to 90–95% with full immunization. Therefore, we introduce as an alternative variable the proportion of the population that is already fully vaccinated. Column two contains the proportion of fully vaccinated individuals. Our results point in the direction of clinical studies and indicates that full vaccinations show a stronger and more significant correlation than the effect after the first dose. Therefore, while a significant effect can already be identified with the first dose, it increases with full vaccination. After full vaccination, the effect on the change in the reproduction rate is about twice as large.^4^

Since data availability for full vaccination protection is poorer than vaccinated at least once, we obtain this result based on a slightly modified smaller sample.

Further, we test for a nonlinear effect of vaccination. If the proportion of vaccinated individuals (and those who have recovered) increases in a region, the disease can no longer spread so quickly there. Above a certain vaccination rate, even herd immunity is achieved. Theoretically, we can therefore assume that as the vaccination rate increases, the marginal effect on the reproduction rate decreases. We include the squared term of vaccinations to test for this non-linearity. The results are presented in column 3 for the proportion of vaccinated individuals and in column 4 for fully vaccinated individuals. While in the beginning the effect of vaccination is stronger, it decreases as the proportion of vaccinated individuals increases.

In the model presented earlier, it is assumed that the effects of the individual measures influence each other. For example, it can be assumed that above a certain vaccination rate or herd immunity, other measures are no longer necessary and have no effect. To examine how the mix of COVID-19 measures behaves with an increase in the vaccination rate in the population, we next test interactions of Tests _t-1_ and Stringency index _t-1_ with the vaccination rate. The estimates of the equations for this are given in columns five and six of Table 2. However, of particular interest are the marginal effects of Tests _t-1_ and Stringency index _t-1_ for specific values of the vaccination rate, which are shown in Table 3. While the effects of the stringency variable remain significant throughout and remain roughly the same in terms of effect size, the marginal effects of the test variable reveal interesting findings. The effects for Tests _t-1_ become smaller with increasing vaccination rates and the significance decreases. At vaccination rates above 50%, the results even become insignificant. This indicates that, especially with low vaccination rates, a systematic testing strategy can help to identify infected individuals and thus break the chains of infection. With a higher vaccination rate, systematic testing is shown to be less effective, as a larger proportion of the population is protected by vaccination. In general, our results are in line with previous studies which found that vaccinations had gradually contributed to the suppression of COVID-19 transmission (Ge et al., 2021; Rodríguez et al., 2021). Our results show that this still holds even when controlling for the effects of testing and lockdown strategies.

**Table 3:** Marginal effects

at Vaccinations _t-7_ (%)	Tests _t-1_		Stringency index _t-1_	
	dy/dx	z-statistic	dy/dx	z-statistic
0	− 0.00033^***^	− 4.34	− 0.00029^***^	− 5.76
5	− 0.00031^***^	− 4.66	− 0.00029^***^	− 6.49
10	− 0.00030^***^	− 4.80	− 0.00030^***^	− 7.15
15	− 0.00029^***^	− 4.63	− 0.00030^***^	− 7.60
20	− 0.00028^***^	− 4.19	− 0.0003^***^	− 7.68
25	− 0.00026^***^	− 3.61	− 0.00031^***^	− 7.39
30	− 0.00025^***^	− 3.03	− 0.00032^***^	− 6.87
35	− 0.00024^**^	− 2.53	− 0.00032^***^	− 6.26
40	− 0.00023^**^	− 2.11	− 0.00032^***^	− 5.66
45	− 0.00021^*^	− 1.77	− 0.00033^***^	− 5.12
50	− 0.00020	− 1.49	− 0.00033^***^	− 4.66
55	− 0.00019	− 1.26	− 0.00034^***^	− 4.26
60	− 0.00018	− 1.07	− 0.00034^***^	− 3.92
65	− 0.00016	− 0.91	− 0.00035^***^	− 3.63
70	− 0.00015	− 0.77	− 0.00035^***^	− 3.38
75	− 0.00013	− 0.66	− 0.00035^***^	− 3.16

It should be noted that case finding has shown strong changes during our study period, rising and falling in waves. Further, the proportion of vaccinated in the population has grown over time. In order to account for the changes in case finding, and to be able to isolate the effect of vaccination from the effect of time we perform further estimations including monthly fixed effects. Since the vaccination variable is highly correlated with time, estimating the model with time fixed effects in its original form is not possible. We therefore transform the vaccination variable and generate categorial variables based on a certain vaccinated proportion of the population, i.e. the variable Vaccinations 30 takes the value 1 if at least 30% of the population is vaccinated. The results including time fixed effects and a proportion of vaccinated of 30, 40 and 50% are shown in Table 4 and confirm our previous findings. When additionally controlling for the time trend the effects of tests and state stringency stay robust in terms of effect size and significance. Furthermore, the approach with categorial vaccination variables allows us to specify the effect of vaccination in more detail. While no effect is observed for 30% of the vaccinated population, the expected negative effect is observed for a proportion of more than %. The dummy for a proportion above 50% again shows an insignificant result. However, this may be due to the very small number of cases (less than 2%) that reach this value in the observation period.

**Table 4:** Fixed-effects Results with time fixed effects and categories of vaccinated population

	(1)	(2)	(3)	(4)	(5)	(6)
	ΔRR	ΔRR	ΔRR	ΔRR	ΔRR	ΔRR
Tests _t-1_	− 0.00041^***^ (− 6.126)	− 0.00040^***^ (− 5.986)	− 0.00041^***^ (− 6.008)	− 0.00102^***^ (-9.789)	− 0.00106^***^ (− 10.113)	− 0.00098^***^ (− 9.262)
Vaccinations 30 _t-7_	0.00133 (1.003)		− 0.00029^***^ (− 4.811)		− 0.00016^***^ (− 4.380)	− 0.00015 (− 1.196)
Vaccinations 30 (full) _t-7_		− 0.00034 (− 0.149)				
Vaccinations 40 _t-7_			− 0.00326^**^ (1.965)			
Vaccinations 40 (full) _t-7_				− 0.00568^**^ (− 1.994)		
Vaccinations 50 _t-7_					0.00174 (0.653)	
Vaccinations 50 (full) _t-7_						0.00403 (1.429)
Stringency index _t-1_	− 0.00026^***^ (− 6.743)	− 0.00025^***^ (− 6.131)	− 0.00026^***^ (− 6.380)	− 0.00037^***^ (− 8.839)	− 0.00040^***^ (− 9.207)	− 0.00035^***^ (− 8.255)
Time fixe effects	Yes	Yes	Yes	Yes	Yes	Yes
*R*^*2*^ within	0.068	0.088	0.069	0.089	0.068	0.089
Countries	37	36	37	36	37	37
Obs	3810	3366	3810	3366	3810	3810

*t* statistics in parentheses

*p < 0.10, **p < 0.05, ***p < 0.01

Next, we examine the individual policy measures contained in the stringency index. We divide the index into its individual components and test these simultaneously. Table 5 presents the results. While the test and vaccination variables remain highly significant, only three of the nine index components show significant results. School and workplace closures show the strongest effects, which are very similar in terms of size and significance. A one-unit increase in the indices—for example, from 0 (no measures) to 1 (closures recommend—is associated with a 0.4% decrease in the reproduction growth rate. A one-unit increase in restrictions on internal movement goes along with a 0.1% decrease, respectively. This confirms previous findings that point at significant effects of non-pharmaceutical interventions like school (Banholzer et al., 2020; Ge et al., 2021; Sharma et al., 2021) and workplace closures (Askitas et al., 2021; Banholzer et al., 2020), and which argue that general lockdowns (Flaxman et al., 2020; Noland, 2021) and internal movement restrictions (Vannoni et al., 2020) reduce mobility and lower the infection rate. However, in most of these papers, non-pharmaceutical interventions were considered in isolation and other instruments were largely disregarded. By contrast, our analysis examines the stringency measures in context with other policy measures, providing a more comprehensive picture.

**Table 5:** The Effects of COVID-19 Response Indicators

	(1)	(2)	(3)
	ΔRR	ΔRR	ΔRR
Tests _t-1_	− 0.00043^***^ (− 6.516)	− 0.00045^***^ (− 6.776)	− 0.00044^***^ (− 6.570)
Vaccinations _t-7_	− 0.00014^***^ (− 4.377)	− 0.00015^***^ (− 4.874)	− 0.00013^***^ (− 4.078)
School closures _t-1_	− 0.00364^***^ (− 6.165)		− 0.00367^***^ (− 6.174)
School closures (1, 2, 3) _t-1_		0.00789 (1.125)	
School closures (2, 3) _t-1_		− 0.00004 (− 0.042)	
School closures (3) _t-1_		− 0.00654^***^ (− 7.896)	
Workplace closures _t-1_	− 0.00449^***^ (− 6.293)	− 0.00433^***^ (− 6.081)	
Workplace closures (1, 2, 3) _t-1_			0.00124 (0.216)
Workplace closures (2, 3) _t-1_			− 0.00288^*^ (− 1.877)
Workplace closures (3) _t-1_			− 0.00488^***^ (− 6.268)
Cancelled public events _t-1_	0.00188 (1.530)	0.00112 (0.906)	0.00127 (0.992)
Restrictions on gatherings _t-1_	0.00095 (1.160)	0.00017 (0.195)	0.00077 (0.899)
Public transport closures _t-1_	− 0.00003 (− 0.029)	− 0.00031 (− 0.276)	0.00001 (0.005)
Stay-at-home requirements _t-1_	− 0.00032 (− 0.380)	− 0.00020 (− 0.230)	− 0.00005 (− 0.053)
Restrictions on internal movement _t-1_	0.00109^*^ (1.664)	0.00091 (1.353)	0.00090 (1.354)
International travel controls _t-1_	0.00062 (0.662)	0.00028 (0.299)	0.00059 (0.621)
Public information campaigns _t-1_	− 0.00330 (− 0.487)	− 0.00076 (− 0.112)	− 0.00217 (− 0.319)
*R*^2^ within	0.042	0.049	0.043
Countries	37	37	37
Obs	3794	3794	3794

*t* statistics in parentheses

*p < 0.10, **p < 0.05, ***p < 0.01

To shed more light on the effects of the highly significant index components, we generate categorial dummies for the different values of the ordinal index scale. For example, the dummy School closures (1, 2, 3) _t-1_ is 1 for the categories 1 (closure recommended), 2 (closure required on some levels), and 3 (closure required on all levels), and 0 for 0 (no measures). The dummy School closures (2, 3) _t-1_ is 1 for the categories 2 and 3 and 0 for the categories 0 and 1. The dummy School closures (3) _t-1_ is 1 for the category 3 and 0 for the categories 0, 1 and 2. This allows us to identify whether the individual policy measures mapped by each level of the indices have a significant effect. The results are presented in columns two and three of Table 5. While the dummies including the first and second categories are not significant, the dummy representing required closures on all levels shows a highly significant result. While the general recommendation to close or open schools with alterations and selective closures on only some levels show no significant effects, a stronger restriction in the form of required closures for all school levels is significantly related with reductions in the reproduction rate. The situation is similar for job restrictions. Again, the inclusion of the lightest restrictions in the form of a recommendation to close and to work from home shows no significant effect. Only stricter restrictions such as required closures or a firm rule to work from home for some sectors or categories of workers (category 2) and required closures for all but essential workplaces (category 3) lead to a significant correlation with a reduction in infection rates. While the dummy that includes the second category only shows significance at the 90% level, the strictest category is highly significant and shows a stronger effect in terms of magnitude.

Overall, our results show that a mix of different measures is necessary to combat the pandemic. Until vaccination has progressed to the point where herd immunity has been achieved or where there is no option to achieve herd immunity, the pandemic can also be controlled by non-pharmaceutical interventions and testing, quarantine, and isolation strategies. This is significant given the possibility of new mutations against which current vaccines show no or less efficacy. Under the given circumstances, (Moore et al., 2021) show for the case of the UK that vaccination alone is insufficient to contain the pandemic, and that its effect is strongly contingent upon the precise vaccine properties and population uptake.

## Concluding remarks

An essential element of the policy measures to curb the COVID-19 virus is contact prevention. Lockdown strategies to combat the pandemic have a substantial impact on social and economic life and involve immense economic costs. Tools such as testing and vaccination strategies can allow societies more liberties by enabling restrictions to be relaxed in public and private. Our study examines the effects of these control measures on the reproduction rate of COVID-19.

We set up a theoretical framework to model the effects of policy measures on the reproduction rate. Based on this, we perform a panel analysis using daily data covering 37 OECD countries from March 1, 2020 to May 20, 2021 to assess the relative effectiveness of testing, vaccinations and non-pharmaceutical government response measures. Our estimates provide empirical evidence on the influence of testing, vaccination and specific non-pharmaceutical interventions on the dynamic of the reproduction rate. The simultaneous and data-driven consideration of the instruments provides a comprehensive picture and helps to understand the effectiveness of these measures in the current fight against the pandemic under consideration of different conditions and strategies within countries.

For three of our nine NPIs, we identify an estimated relative reduction in the reproduction rate with workplace and school closures showing the strongest effects. A more differentiated analysis shows that the significant effects can only be identified in the case of complete closures. Restricting internal movement likewise reduces contacts and lowers the reproduction rate. With regard to the timing of introduction of social distancing, the lockdown effect is shown to be strongest immediately, decreases with time and approaches zero over time indicating that the adoption time of NPIs and the adjustment to current conditions is important. An early response is critical to counteracting the virus most effectively, as the effect is visible and strongest immediately after introduction.

As expected, our results confirm that while vaccinations can mitigate the pandemic, there are significant differences between first-dose vaccinations and full vaccination protection. Further, we can identify diminishing marginal effects suggesting that initially, vaccinations have a strong effect; a certain level of vaccination is required to better control the pandemic. Once this level is reached, additional vaccinations generate only reduced effects. This points to a release of lockdown measures once sufficient vaccines are available and natural herd immunity is achieved. Finally, we find a significant effect for testing. However, a closer look at the interaction with vaccinations shows that this effect only remains significant up to a vaccination rate of about 50%. For as long as vaccines are not available in sufficient quantity or population take up is insufficient, a test, trace, and isolate strategy can help keep the pandemic under control.

Our analysis is limited by the type of data utilized. First, the results are based on PCR test statistics. Unfortunately, the number of rapid and self-tests is unknown for the country panel. Therefore, when using the number of PCR tests, we are aware that the effect of testing is relatively strong because the number of rapid and self-tests is much higher than that of the PCR tests. Second, our analysis does not explicitly account for the recent increase in viral variants. The differentiation of variants has only recently been included in the statistics and data is still not widely available across countries. Once this is remedied, future studies could take the differentiation of variants into account and examine whether virus variants change the effectiveness of the instruments.

## Appendix

See Tables 6 and 7

**Table 6:** Variables definitions and sources

Variable	Time	Definition	Source
Reproduction rate	Ddaily	Reproduction rate	Our World in Data COVID-19 dataset, (Arroyo-Marioli et al., 2021)
Tests	Daily	New tests smoothed per thousand	Our World in Data COVID-19 dataset, (Hasell et al., 2020)
Vaccinations	Daily	Individuals vaccinated per hundred; individuals fully vaccinated per hundred	Our World in Data COVID-19 dataset, (Ritchie et al., 2020)
Stringency index	Daily	Composite measure based on nine response indicators including school closures, workplace closures, and travel bans, rescaled to a value from 0 to 100 (100 = strictest) If policies vary at the subnational level, the index is shown as the response level of the strictest sub-region	Oxford COVID-19 Government Response Tracker, Blavatnik School of Government, (Hale et al., 2021)
School closures	Daily	0—no measures 1—closures recommended or all schools open with alterations resulting in significant differences compared to non-COVID-19 operations 2—closures required (only some levels or categories, e.g. only high school or only public schools) 3—closures required at all levels	Oxford COVID-19 Government Response Tracker, Blavatnik School of Government, (Hale et al., 2021)
Workplace closures	Daily	0—no measures 1—closures recommended (or recommendation to work from home) or all businesses open with alterations, resulting in significant differences compared to non-COVID-19 operation 2—closures required (or work from home) for some sectors or categories of workers 3—closures required (or work from home) for all but essential workplaces (e.g., grocery stores, doctors)	Oxford COVID-19 Government Response Tracker, Blavatnik School of Government, (Hale et al., 2021)
Cancelled public events	Daily	0—no measures 1—cancellation recommended 2—cancellation required	Oxford COVID-19 Government Response Tracker, Blavatnik School of Government, (Hale et al., 2021)
Restrictions on gatherings	Daily	0—no restrictions 1—restrictions on very large gatherings (the limit is above 1000 people) 2—restrictions on gatherings between 101–1000 people 3—restrictions on gatherings between 11–100 people 4—restrictions on gatherings of 10 people or less	Oxford COVID-19 Government Response Tracker, Blavatnik School of Government, (Hale et al., 2021)
Public transport closures	Daily	0—no measures 1—closures recommended (or significant reduction in frequency /route/means of transport available) 2—closures required (or most citizens banned from using it)	Oxford COVID-19 Government Response Tracker, Blavatnik School of Government, Hale, et al. (2021) (Hale et al., 2021)
Stay-at-home requirements	Daily	0—no measures 1—recommendation not to leave house 2—requirement not to leave house with exceptions for daily exercise, grocery shopping, and “essential” trips 3—requirement not to leave with minimal exceptions (e.g. permission to leave once a week, or only one person may leave at a time, etc.)	Oxford COVID-19 Government Response Tracker, Blavatnik School of Government, (Hale et al., 2021)
Restrictions on internal movement	Daily	0—no measures 1—recommendation not to travel between regions/cities 2—internal movement restrictions in place	Oxford COVID-19 Government Response Tracker, Blavatnik School of Government, (Hale et al., 2021)
International travel controls	Daily	0—no restrictions 1—screening arrivals 2—quarantine for arrivals from some or all regions 3—ban on arrivals from some regions 4—ban on all regions or total border closure	Oxford COVID-19 Government Response Tracker, Blavatnik School of Government, (Hale et al., 2021)
Public information campaigns	Daily	0—no COVID-19 public information campaign 1—public officials urge caution about COVID-19 2—coordinated public information campaign (e.g. across traditional and social media)	Oxford COVID-19 Government Response Tracker, Blavatnik School of Government, (Hale et al., 2021)

**Table 7:** OLS and System GMM estimations

	(1) OLS ΔRR	(2) FE ΔRR	(3) SYS-GMM ΔRR
Tests _t-1_	− 0.00005^**^ (− 2.089)	− 0.00041^***^ (− 6.180)	− 0.00016^***^ (− 9.278)
Vaccinations _t-7_	− 0.00012^***^ (− 5.892)	− 0.00016^***^ (− 5.435)	− 0.00023^***^ (− 11.326)
Vaccinations (full) _t-7_			
(Vaccinations _t-7_)^2^			
(Vaccinations (full) _t-7_)^2^			
Stringency index _t-1_	− 0.00008^***^ (− 3.895)	− 0.00030^***^ (− 7.679)	− 0.00038^***^ (− 13.956)
*R*^2^	0.014		
*R*^2^ within		0.028	
AR(2) test			1.80 (0.071)
Hansen test			36.11 (0.325)
Instruments			37
Countries	37	37	37
Obs	3810	3810	3810

*t* statistics in parentheses

**p* < 0.10, ***p* < 0.05, ****p* < 0.01

## Footnotes

1 See e.g. (Nishiura and Chowell 2009).

2 The countries included in the analysis are Australia, Austria, Belgium, Canada, Chile, Colombia, Costa Rica, Czech Republic, Denmark, Estonia, Finland, France, Germany, Greece, Hungary, Iceland, Ireland, Israel, Italy, Japan, Latvia, Lithuania, Luxembourg, Mexico, the Netherlands, New Zealand, Norway, Poland, Portugal, Slovak Republic, Slovenia, Spain, Sweden, Switzerland, Turkey, the United Kingdom and the United States.

3 We also run OLS estimation and System GMM estimation using internal instruments for the independent variables to counteract possible endogeneity in the model. The results are presented in Table 7 in the Appendix and remain robust to the FE results. Further, we apply the Durbin-Wu-Hausman endogeneity test to test if our independent variables are exogenous. With a p-value of 0.20 we cannot reject the H0 hypothesis of exogeneity. Therefore, in the following we perform the estimations based on the fixed-effects model.

4 Although our results generally confirm the effectiveness results of clinical studies, the degree of increase from first to full vaccination differs. On the one hand, the differences between our results and clinical studies may result from the fact that these are based on two different methodological approaches. Our analysis is based on a panel analysis with country data partly resulting from average observations. Individual effects, as they are found in clinical studies, cannot be measured directly. On the other hand, we consider a mix of several pandemic measures at the same time. In clinical studies, however the effectiveness of the vaccination is considered in isolation, other factors such as testing or lockdown measure are left out. Finally, our study does not explicitly specify which vaccine was used; all vaccines are considered cumulatively.
